# Assessing the reliability of automatic sentiment analysis tools on rating the sentiment of reviews of NHS dental practices in England

**DOI:** 10.1371/journal.pone.0259797

**Published:** 2021-12-15

**Authors:** Matthew Byrne, Lucy O’Malley, Anne-Marie Glenny, Iain Pretty, Martin Tickle

**Affiliations:** Faculty of Biology, Division of Dentistry, Medicine and Health, School of Medical Sciences, University of Manchester, Manchester, England, United Kingdom; European Commission, ITALY

## Abstract

**Background:**

Online reviews may act as a rich source of data to assess the quality of dental practices. Assessing the content and sentiment of reviews on a large scale is time consuming and expensive. Automation of the process of assigning sentiment to big data samples of reviews may allow for reviews to be used as Patient Reported Experience Measures for primary care dentistry.

**Aim:**

To assess the reliability of three different online sentiment analysis tools (Amazon Comprehend DetectSentiment API (ACDAPI), Google and Monkeylearn) at assessing the sentiment of reviews of dental practices working on National Health Service contracts in the United Kingdom.

**Methods:**

A Python 3 script was used to mine 15800 reviews from 4803 unique dental practices on the NHS.uk websites between April 2018 –March 2019. A random sample of 270 reviews were rated by the three sentiment analysis tools. These reviews were rated by 3 blinded independent human reviewers and a pooled sentiment score was assigned. Kappa statistics and polychoric evalutaiton were used to assess the level of agreement. Disagreements between the automated and human reviewers were qualitatively assessed.

**Results:**

There was good agreement between the sentiment assigned to reviews by the human reviews and ACDAPI (k = 0.660). The Google (k = 0.706) and Monkeylearn (k = 0.728) showed slightly better agreement at the expense of usability on a massive dataset. There were 33 disagreements in rating between ACDAPI and human reviewers, of which n = 16 were due to syntax errors, n = 10 were due to misappropriation of the strength of conflicting emotions and n = 7 were due to a lack of overtly emotive language in the text.

**Conclusions:**

There is good agreement between the sentiment of an online review assigned by a group of humans and by cloud-based sentiment analysis. This may allow the use of automated sentiment analysis for quality assessment of dental service provision in the NHS.

## Introduction

Online reviews are increasingly being used in order to allow patients to express their views about the quality of their health care [1]. The ubiquity of online media and the ease at which reviews can be left for healthcare providers means that online ratings and reviews are used commonly by patients to compare providers; the immediacy at which reviews can be left may provide physicians with quick feedback on the care they provide but it is unclear as to how reliable this source of data is [2]. A study by Holliday et al. [3] demonstrates that patients value the use of online ratings and feel that experience ratings should be publicly available; physicians however were less open to this data being used to assess the quality of care they provide.

NHS.uk (formerly NHS Choices) is a website that collates data for National Health Service (NHS) healthcare services in the UK. This website allows patients to rate their healthcare provider using a 5-star system and free text comments. A study of these data by Brookes and Baker [4] shows that comments could be assessed to provide generalisable insights into the quality of care, and that dental practices are frequently reviewed by patients. There are large number of reviews that are left by patients on their dental practices means that this may be a useful source of information for the assessment of patients’ experiences in and the quality of primary dental care.

Assessing the sentiment of a large body of reviews manually is time consuming and costly. Furthermore, no official body is currently using online reviews on a large scale in the UK to assess the quality of care that is provided. There are a number of cloud-based solutions to automatic sentiment analysis which can allow large sample sizes to be analysed and categorised in minutes [5]. These analysis tools have been developed for the service industry and for online businesses to assess the satisfaction of their customers. It is not yet known if these sentiment analysis tools are sufficiently accurate to reliably assess the views of the English public with regards to their dental care. A study by Gray et al. [6] has shown that online ratings have poor association with traditional quality measures, but had a statistically significant association with Patient Reported Experience Measures (PREMs).

The most widely used PREM for NHS dental patients in the UK is the Friends and Family Test (FFT); this uses a 5-point Likert scale to assess patient sentiment. Each dental practice is expected to submit at least five FFT outcomes to the NHS to contribute to a database which is reported nationally [7]. Despite a contractual obligation for practices to collect patient reported experience using the FFT, adherence to this is poor. Furthermore, the most common way that this is completed is on paper in the dental practice, suggesting a high risk of patients not reporting their true opinions.

This research aims to assess the reliability of automatic sentiments analysis methods. If such methods are reliable, this would allow for novel methods of assessing the sentiment of dental patients with the potential for this to be used as a measure of quality in primary dental care.

## Aim

This study aims to assess the reliability at which cloud based automatic sentiment analysis tools assess and assign the sentiment of review text that is posted for dental practices on the NHS.uk website.

## Methods

A script written in the Python 3 programming language was used to mine data from the NHS.uk website. The Beautifulsoup Python module [8] was used to parse the review text, star rating and date posted of reviews into a.csv file. 15800 reviews taken from all available dental practice webpages on the NHS website from March 2019 to February 2020. Each review was assigned a unique identifier and converted into a.txt file.

Amazon Comprehend is a commonly used and widely available cloud-based sentiment analysis tool. It can assess the linguistic content of text and assign a score of positive, negative neutral or mixed sentiment to a body of text. Reviews were parsed into individual.txt files and synced to an Amazon Simple Storage Solution (SSS) bucket. This bucket was synced to the Amazon Comprehend DetectSentiment Application Protocol Interface (ACDAPI) using the Amazon Lambda interface, allowing for each filed to be analysed and a have a score assigned to it, which was output as a.csv file. These.csv files were then synced back to a main computer using the Amazon Command Line Interface (CLI), and reassociated with the review text.

All reviews were assigned an overarching sentiment of positive, negative neutral or mixed (Table 1). Alongside the labelled output, each text is provided with a certainty score in each of the 4 categories as a ratio of 1, between 0–1 for each of these 4 named categories. For the purposes of this study, only the categories are considered to allow for comparison between different measures [9].

**Table 1:** Definitions of different sentiment scores.

Sentiment assigned	Description
Positive	Sentiment of text is positive reflecting good experiences or actions
Negative	Sentiment of the Text is negative representing bad experiences or actions
Neutral	The review text contains language with little or no sentiment
Mixed	There is a mixture of positive and negative elements in the text

As this study was assessing the agreement between different raters on a categorical scale, Cohen’s Kappa was used [10]. Initial analysis of these suggested that the review was heavily weighted towards positive and negative reviews with few mixed or neutral reviews. For samples with unequal weighting to each category, sample size for Cohen’s kappa is determined according the predicted proportion of each category of review [11]. The overall output of the ACDAPI was assessed, giving a proportion of 0.82,0.13,0.04,0.01 for Positive, Negative, Neutral and Mixed respectively. These proportions were used with the sample size for Cohen’s Kappa calculator function in the R programming language, with k1 = 0.6, k0 = 0.4 and power = 0.9, giving a sample of 270 reviews.

A random number generator was used to select 270 reviews for assessment by the human reviewers. Three reviewers (MT, LO, AMG, all researchers, 1 dentally qualified) independently assessed the reviews. Each were blinded to the star rating and computer assigned sentiment analysis score. Using an Excel spreadsheet, the reviewers assigned a score of positive, negative, neutral, or mixed to each review according to the criteria in Table 1. A Kruskall Wallis test was used to test for significant differences between these 3 reviewers. Fleiss Kappa [12] was used to assess the agreement the Inter-rater reliability of the three reviewers using IBM SPSS version 25. The results of the 3 independent human reviews were pooled to give a consensus of the sentiment of the reviews. Where there were disagreements, these were discussed until the group was happy that the rating sufficiently described the sentiment of the review.

As the ACDAPI is a machine learning cloud system that relies on a massive, increasing dataset, it was unknown if there would be any changes to outputs as the model learned over time. To test this, 2 months after the initial use of the ACDAPI the sample of 270 records were retested. This demonstrated no change in the output or certainty scores.

Sentiment analysis APIs provided by Google Cloud services and by Monkeylearn were also tested. Unlike the ACDAPI, these are unable to natively export their sentiment analysis to a.csv output, and are not as capable of handling ‘big data’. Due to time constraints, the sample of 270 reviews rated by human reviewers were manually uploaded to each system to record their relative output.

Kappa statistic was then used to compare the pooled sentiment assigned by the reviewers to the sentiment assigned by the ACDAPI, Monkeylearn and Google sentiment analysis APIs. Mann-Whitney U tests were performed to test for significant differences between the pooled reviews and each sentiment type. Polychoric evaluation was used to further verify concordance strength [13].

A qualitative assessment of the reviews in which there was a disagreement between the output of the ACDAPI and human reviewers was performed by MB to assess the reasons for disagreements. This was done through coding of the text and synthesis of themes.

## Results

The proportion of each sentiment in the random sample closely reflected that of the full text (**Table 2**).

**Table 2:** Distribution of assigned sentiment scores in whole dataset and sample.

	Whole dataset (Proportion of response/1) n = 15800	Sample (Proportion of response/1) n = 270
Positive	0.82 (n = 12956)	0.83 (n = 224)
Negative	0.13 (n = 2054)	0.12 (n = 32)
Neutral	0.04 (n = 632)	0.04 (n = 11)
Mixed	0.01 (n = 158)	0.01 (n = 3)

**Fig 1:**
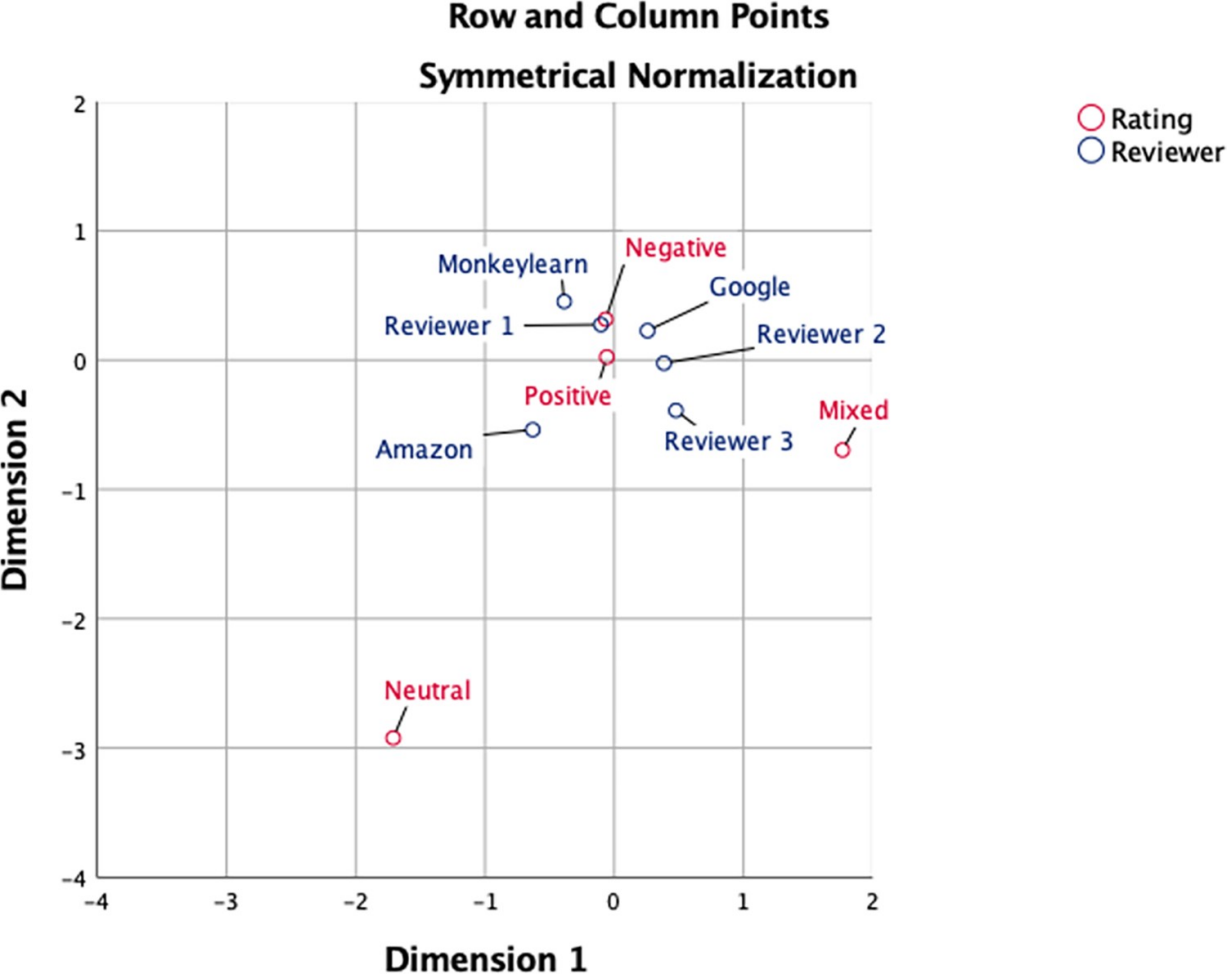
Correspondence analysis plot showing relation between reviewers and ratings. Proximity of reviewer to rating suggests tendency of reviewer towards that rating. In the sample of 270 reviews there were 33 disagreements between the ACDAPI and the reviewers. n = 16 errors were due to emotive syntax discrepancies, n = 10 were due to misappropriation of the strength of conflicting emotions and n = 7 were due to a lack of overtly emotive language in the text.

**Fig 1** Displays the ratings of each of the reviewers, alongside that of the pooled reviewers, demonstrating a propensity towards positive revies and similar levels of positive and negative ratings.

Fleiss Kappa was used to compare the three human reviewers. This showed an overall kappa of 0.836 (**Table 3**). According to Landis and Koch [14], this constitutes almost perfect agreement. However, disagreements were proportionately much higher in the mixed and neutral categories.

**Table 3:** Fleiss Kappa comparing three human reviewers.

Fleiss Kappa Reviewer 1 vs 2 vs 3 and Kappa for Individual Categories							
Rating Category	Conditional Probability	Kappa	Asymptotic Standard Error	Z	P Value	Lower 95% Asymptotic CI Bound	Upper 95% Asymptotic CI Bound
Overall	N/A	0.836	0.027	30.828	0	0.783	0.889
Positive	0.98	0.897	0.035	25.537	0	0.828	0.966
Negative	0.911	0.898	0.035	25.563	0	0.829	0.967
Neutral	0.5	0.496	0.035	14.124	0	0.427	0.565
Mixed	0.62	0.595	0.035	16.934	0	0.526	0.664

A Kruskall Wallis test demonstrated no significant difference (2.967 df = 2, p = 0.227) between the ratings supplied by the 3 reviewers, demonstrating that the pooled reviews may be used for further analysis.

Agreement with Cohen’s Kappa between the pooled reviews and the Amazon API had a Kappa value of 0.660 (**Table 4**). This suggests substantial agreement between the human reviewers and the ACDAPI [14]. Of the 270 reviews, 247 were assigned the same sentiment score.

**Table 4:** Cohen’s Kappa comparing Monkeylearn to Human reviewers and Google Cloud Services to human reviewers.

	Cohen’s Kappa Pooled human reviews vs Monkeylearn and Google, with Kappa for Individual Categories							
	Rating Category	Conditional Probability	Kappa	Asymptotic Standard Error	Z	P Value	Lower 95% Asymptotic CI Bound	Upper 95% Asymptotic CI Bound
Amazon vs Pooled Human reviews	Overall	N/A	0.66	0.05	12.262	0	0.562	0.757
	Positive	0.964	0.79	0.061	12.987	0	0.671	0.91
	Negative	0.714	0.672	0.061	11.038	0	0.552	0.791
	Neutral	0.167	0.148	0.061	2.427	0.015	0.028	0.267
	Mixed	0.2	0.185	0.061	3.038	0.002	0.066	0.304
Monkeylearn vs Pooled Human reviews	Overall	N/A	0.728	0.052	14.101	0	0.606	0.83
	Positive	0.966	0.808	0.061	13.283	0	0.689	0.928
	Negative	0.842	0.816	0.061	13.412	0	0.697	0.936
	Neutral	1	1	0.061	16.432	0	0.881	1.19
	Mixed	0	-.0.33	0.061	-0.534	0.593	-0.152	0.087
Google vs Pooled Human Reviews	Overall	N/A	0.706	0.049	14.52	0	0.61	0.801
	Positive	0.965	0.828	0.061	13.599	0	0.708	0.947
	Negative	0.842	0.816	0.061	13.412	0	0.697	0.936
	Neutral	0	-0.002	0.061	-0.03	0.976	-0.121	0.117
	Mixed	0.188	0.136	0.061	2.24	0.025	0.017	0.256

Breaking down the individual categories, there was good agreement on reviews assigned as negative (k = 0.672) and even better agreement on reviews assigned as positive (k = 0.790). There was poor agreement on reviews assigned as neutral (k = 0.148) or mixed (k = 0.185), to the degree that this could not be differed from random chance.

The Monkeylearn sentiment analysis API gave compared to the pooled review gave a kappa value of 0.728. The Google Cloud sentiment analysis API compared to the pooled reviews yielded a Kappa of 0.706. This suggests slightly better agreement between these APIs and the Amazon, however all still fit within the ‘Substantial Agreement’ range set out by Landis and Koch [14]. Mann-Whitney U tests demonstrated no significant difference between the Pooled and Amazon (p = 0.434), Pooled and Google (p-0.659) or Pooled and Google (p = 0.186) groups.

**Table 5** demonstrates Polychoric evaluation of the ratings of each raters, demonstrating good levels of agreement between each of the groups.

**Table 5:** Polychoric correlation matrix demonstrating agreement between each group.

	Pooled Human raters	Amazon ACDAPI	Monkeylearn	Google
Pooled Human raters	1.000	0.782	0.831	0.783
Amazon ACDAPI	0.782	1.000	0.775	0.704
Monkeylearn	0.831	0.775	1.000	0.775
Google	0.783	0.704	0.775	1.000

**Fig 1** displays a correspondence analysis plot for these data. This demonstrates clustering of all reviewers around Positive and Negative ratings, an increased tendency for ACDAPI(amazon) to rate a review as ‘Neutral’.

The syntax of reviews had an impact upon how well the reviews were assessed. Emotive words such as ‘pain’, ‘traumatic’, ‘scared’ would be assessed as negative, but the context of the whole review may show this is not the case. For example, a review that describes how visiting the dentist is normally traumatic, but in this circumstance was good, may be rated as negative due to the strong negative term used. In these instances, reviews were usually wrongly assigned as positive or negative.

Conversely, where a person leaving a review does so without terms that are particularly emotive, a neutral score was likely to be given to the text. In almost all situations, the human reviewers were able to better understand the implication of the review and assign an appropriate sentiment to this. In these situations, the ACDAPI assigned a neutral score, but the human reviewers assigned a positive, negative, or mixed response.

Finally, where there were truly mixed reviews in which both negative and positive statements and terms that were used, the relative strength of the emotion was not well assessed. For example, a review that complains about having to wait for some time, but then highly praises the quality of care may be assigned as negative but may be assigned as mixed or positive by the human reviewers. This was the most common error that was assessed. **Table 6** demonstrates examples of each error type. Whilst the text of reviews is on an online public resource, the example text for each error type has been synthesised from text corpuses to protect the identity of the patients and staff of the associated practices [15].

**Table 6:** Examples of 3 main error types demonstrated between the ACDAPI and Human reviewer. Sample text synthesised to prevent identification of patients or staff.

ACDAPI Sentiment	Human Assigned Sentiment	Review text	Error Type
Negative	Positive	My daughter was frightened about the prospect of 2 planned extractions. The dentist and nurse were kind and patient. Together they made a traumatic experience easier for my daughter	Syntax error: Emotive terms such as frightened, traumatic experience are not considered in context of positive review
Neutral	Positive	Always has time to talk about my dental hygiene in depth. The dentist could relate dental problems to other health issues and recommend treatments. If I have any problems I can come back before the recommended 6 months.	Language used is not overtly emotive. Satisfaction is easily inferred by human reviewers.
Negative	Mixed	My dentist is excellent, but he is the only reason that I attend this practice. The reception staff are rude. I cannot see why this is not being addressed after I have complained.	Strength of conflicting emotions. This patient feels strongly that their dentist is good but that the other staff are poor. ACDAPI overestimates the negative compared to the human reviewers.

## Discussion

The use of online reviews for the assessment of quality in primary dental care is a novel approach to quality assessment and measurement. The ability of automated solutions to the assessment of quality on a massive scale may provide policy makers, commissioners, and dentists with further data to show how services are perceived and how they may be improved.

The strengths of this study are the use of a large data set, and the use of several independent reviewers to assess the sentiment of reviews. Cohen’s kappa is a well-used and reliable measure of agreement that has previously been used to assess the agreement of different raters of patient statements of satisfaction [16].

Whilst the Amazon API was the main sentiment analysis method used for the review data this showed a lower kappa score than the Google and MonkeyLearn APIs over the same dataset when compared to the human reviewers. All the results fell within the 0.6–0.8 range suggesting substantial agreement. Agreement between the reviewers was very good >0.8 and by pooling the result. There were no disagreements of the overall sentiment in the none of the pooled results i.e., between the human reviewers there were only disagreements between reviews being mixed or negative/positive, in no circumstance was a review rated as positive by a reviewer and negative by another. Given this almost perfect agreement between the human reviewers, is further development required for automatic sentiment analysis to reach the abilities of human reviewers to detect the sentiment of reviews. Su et al. [17] describe a method by which automatic and human sentiment analysis can be combined through supervised automation in order to improve the reliability at which automated responses can be attained. This, however, would require the construction of a bespoke machine learning algorithm for sentiment analysis. In this study only off the shelf solutions were assessed, this was due to the balance of cost and time pressures against overall accuracy. Construction of a bespoke sentiment analysis tool would be technically possible and may better represent the sentiments of the UK perspective. However, the benefit of the amazon API is the speed at which it can be used and the fact that this has already been trained by a massive dataset. Given that the ACDAPI is relatively inexpensive to run, a bespoke supervised automation approach may increase the costs of such a system exponentially.

A limitation of this research can be found in the use of Cohen’s kappa to compare the sentiment that is assigned by both human and non-human reviewers. Whilst the assignation of sentiment is intuitive, the lack of clarity as to the exact definition of each sentiment score that the ACDAPI provides means that one cannot explore further the reasons between disagreements. As the sentiment analysis runs from a machine learning algorithm, it is theorised that over time sentiment analysis by the API will more closely approach that of the human reviewers. In the 2 months between the test and retest of the sentiment of the Amazon API there were no changes in the sentiment scores or the output of the files. It is likely that in this period there had been no significant changes in the machine learning algorithm, and it is unknown as to how long such an off the shelf solution would take to improve.

The use of online reviews as a source of patient experience and quality assessment is becoming more prevalent in the literature, however, there have been few studies that look at dental care, and none within the context of NHS dentistry in the UK. A recent study in the United states has compared the contents of online reviews of American dentists from the HealthGrades patient review website to the Consumer Assessment of Healthcare Providers and Systems (CAHPS) survey [18]. This showed that the online review data may be used to assess healthcare quality and explores thematic analysis of reviews. The context of NHS dentistry in the UK differs from the American experience, and this in turn may alter the content of review. The NHS.uk website allows the patient to rate the service that is provided by the practice as opposed to a rating of the individual dentist. As such the ratings provided by patients may be more personal to the individual dentist. The present study adds to the literature by exploring the validity of off-the-shelf sentiment analysis techniques, which may be more easily applied to a translational product for practical use in the NHS. This type of product may have utility to patients in showing how satisfied other patients are with a service, affording them a greater degree of knowledge when selecting services.

Due to the multifaceted and complex nature of quality assessment, further investigation of how well text analysis performs when considered against more traditional metrics of quality, and with more simplistic rating scales such as 5-star rating reviews should be considered. In a study by Gao et al [19], online ratings of medical physicians were compared against existing Quality Metric (QMs). This showed that physicians who were rated as low quality by QMs would receive fewer online reviews and exaggerated the quality of physicians rated highly by traditional QMs. It is argued therefore that such ratings are most useful for assessing the quality of the average quality physician, where they can be better used to discriminate between different providers of a service. The trend of more highly rated practitioners receiving more reviews is also seen by dentists on DocFinder, where they receive 10 times the number of reviews of the dentists rated in the lowest 10% [20]. This may suggest that those practitioners that are proactive in requesting reviews are those that are more likely to provide a high-quality service.

## Conclusions

Automatic sentiment analysis shows good agreement compared to the assessment of human reviewers. For reviews with a clear positive or negative sentiment, automatic sentiment analysis gives ratings that show good agreement to ratings given by humans. This agreement shows the potential to use of online reviews to act as a quality indicator for the care provided by dental professionals. Further work is required to consider the implementation of a quality measure derived from online reviews.

## Supporting information

S1 File (XLSX)
